# Attitudes and Factors Determining the Practice of Routine Medical Checkups in the People of Rawalpindi, Pakistan: A Cross-Sectional Study

**DOI:** 10.7759/cureus.38843

**Published:** 2023-05-10

**Authors:** Faizan Fazal, Hiba Arshad Shahani, Mudassar Fiaz Gondal, Usama Tanveer, Muhammad Haider, Noor Us Sabah, Faizan Shahzad, Mohammad Ebad Ur Rehman

**Affiliations:** 1 Department of Medicine, Holy Family Hospital, Rawalpindi, PAK; 2 Department of Pediatric Surgery, Holy Family Hospital, Rawalpindi, PAK; 3 Department of Orthopaedics, Holy Family Hospital, Rawalpindi, PAK

**Keywords:** annual health checkup, preventive practices, non-communicable disease, comprehensive physical exam, medical screening

## Abstract

Introduction: Routine medical checkup (RMC) is a screening and preventive technique that is implied to detect non-communicable diseases (NCDs). This study aims to assess the awareness in public regarding RMC, the association between education level and level of familiarity regarding RMC, and factors that prevent and encourage the practice of RMC by the public.

Methods: This is a cross-sectional study carried out in Rawalpindi, Pakistan. Health professionals and individuals who refused to consent were excluded from the study. Data was collected using a mixed-mode questionnaire, and convenient sampling was used. The sample size was calculated to be 355 according to the WHO sample size calculator. A total of 356 individuals participated in this study after giving informed consent. Both male and female adults aged 18 or older and residents of Rawalpindi were included in the study. Individuals younger than 18 were excluded.

Results: Among the 356 study participants, 160 (45%) were males, and 196 (55%) were females. The mean age was 27.57±10.027. Among the total participants, 33 (9.3%) individuals had primary-level education, 100 (28.1%) individuals had secondary-level education, and 233 (62.6%) had graduate-level education. A total of 329 (92.9%) participants knew that RMCs could help in early diagnosis and treatment. On the contrary, only 154 (43.3%) people knew that RMCs involve screening all body tissues. Only 329 (92.4%) participants said that they were aware that timely diagnosis through RMC can lead to early treatment. Graduates were generally more aware of different aspects of RMCs, especially in the domains of awareness regarding what an RMC is and that RMC can help in timely diagnosis compared to participants who had primary or secondary level of education (p<0.001). Females had a greater overall awareness of RMCs than males (p<0.001). Graduates were more likely to undergo RMCs than people educated till the primary or secondary level (p<0.001). The most common reason for undergoing RMC was “just concerned about health,” which was selected by 130 (36.5%) participants. The most common reason mentioned by participants for not having an RMC was ''heavy cost,'' mentioned by 104 (29.2%) participants.

Conclusion: Most of the participants of this study were well educated and were students in terms of profession. The majority of the study population knew that RMCs could help in early diagnosis and treatment. Awareness regarding RMCs was linked to educational level. Females had overall better knowledge regarding RMCs than men. The most common reported reason to have an RMC was a health concern, and the most common reported reason for not having an RMC was its high cost.

## Introduction

Non-communicable diseases (NCD) are the leading cause of death and loss of disability-adjusted life-years (DALY) worldwide. According to recent statistics by the WHO, NCDs are responsible for the deaths of 2000 people daily and 40 million people yearly, which is approximately 70% of all deaths globally [1]. Although historically considered as "diseases of affluence," the burden of NCDs has shifted toward low- and middle-income countries in recent years [2]. Major killers of the NCDs are cardiovascular diseases (48%), cancers (21%), chronic respiratory diseases (12%), and diabetes (3.5%) [3]. Globally, the increasing prevalence of NCDs draws public health intervention. The socioeconomic burden of managing these chronic illnesses presents a major challenge to most nations. The disease burden of NCDs can be reduced only by primary prevention and early detection, which can be achieved by RMCs [4]. 

Routine medical checkups (RMCs) are considered effective in preventing illness, promoting health, and reducing morbidity and mortality [5]. Regular health exams and tests can help detect diseases before they progress to a stage where management is less effective and costlier [6]. RMCs can help diagnose chronic NCDs before they cause irreparable damage and permanent disability. During RMCs, some NCDs such as cancer (breast, prostate, cervical), hypertension, and diabetes mellitus can be detected [7]. Studies have also shown in their results that people who undergo regular medical examinations have decreased rates of invasive cancers and mortality [8]. Early detection of cancer through RMCs is crucial to survival rates in cancers. 

Even though RMCs are an effective method of controlling NCDs, their prevalence and awareness are low among the general public. Very few are aware of the benefits of having RMCs and out of those who are aware of the benefits, not everyone deems it necessary to get a RMC. A study in Nigeria revealed that only 79% of those who knew the benefits get RMCs, and out of these only 48% get RMCs frequently, and 52% are not frequent [9]. Pakistan is not exempt from the rising burden of NCDs. As the concept of RMCs is still to be acknowledged by the general public, this research aims to highlight the deficiencies that Pakistan is facing in terms of awareness and frequency of RMCs. 

The reason to conduct this research is to counter NCDs. These diseases are on the high in Pakistan, and people are not economically equipped to counter these diseases so the only way is to prevent them. These diseases can only be prevented if people are medically examined periodically. This research will highlight the deficiency in terms of awareness and RMCs. If people are made aware and are facilitated in getting periodic clinical checkups, it will be a great help in reducing NCDs. This study aims to reveal the frequency, awareness, and practice of RMCs among the general public. This study also aims to find out major factors preventing the practice of RMCs in the general public and to determine the association of demographic variables with the frequency and awareness of RMCs.

## Materials and methods

This is a descriptive cross-sectional study conducted in Rawalpindi, Pakistan. The study duration was six months from July 2022 to January 2023. Convenience sampling (non-probability) was used. The sample size came out to be 355 according to the WHO sample size calculator. A total of 356 individuals participated in this study after giving informed consent. Both male and female adults aged 18 or older and residents of Rawalpindi, Pakistan, were included in the study. Individuals younger than 18 years, health professionals, and individuals who refuse to give consent were excluded from the study. A pilot study was conducted on 30 subjects to assess the internal consistency of the questionnaire. Data was collected by means of a mixed-mode questionnaire that was administered both online and in person. Demographic data were collected. The educational level of participants was divided into three categories: primary education (up to fifth grade), secondary education (up to twelfth grade), and higher secondary (graduate level). Data pertaining to major motivating and preventative factors for getting an RMC was collected. Data will be entered into and analyzed by SPSS Version 28. Descriptive analysis was applied. Mean and percentages were calculated. The chi-square test and Mann-Whitney U test were also applied in this study. The Shapiro-Wilk test was also applied. All personal data will be kept confidential. The anonymity of participants was maintained. This study is self-financed. Ethical approval was taken from the Institutional Review Board of Rawalpindi Medical University, Pakistan. The reference number of the approval letter is PSY-72-46-22. 

## Results

Demographics 

This study gathered data from 356 individuals. Out of which 160 (45%) were males and 196 (55%) were females. The mean age was 27.57±10.02. Education was divided into three categories: 33 (9.3%) individuals had primary-level education, 100 (28.1%) individuals had secondary-level education, and 233 (62.6%) had graduate-level education. The most common occupation among the study participants was "student" with 101 (28.4%) individuals falling into this category. The most common city of residence was Islamabad with 104 (29.2%) study participants belonging to this area.

Pilot study on 30 participants 

A pilot study of 30 participants was done and the internal consistency of the questionnaire was evaluated. Cronbach’s alpha revealed a value of 0.773, which was above the cutoff value of 0.7. Upon performing a Shapiro-Wilk test, age was shown to be non-normally distributed (p<0.001). 

Knowledge of RMC 

Participants were asked about their awareness of RMCs. Only 329 participants (92.9%) were aware that RMCs could help in early diagnosis and treatment. On the contrary, only 154 (43.3%) people knew that RMCs involve screening of all body tissues. A total of 329 (92.4%) participants said that they were aware that timely diagnosis through RMCs can lead to early treatment. Only 205 (57.6%) participants were aware of the screening procedure.

The educational level of participants compared with awareness regarding RMCs 

The level of education was compared with awareness regarding RMCs via a chi-square test. Graduates were generally more aware of different aspects of RMCs (p<0.001), especially in the domains of awareness regarding what an RMC is and that RMC can help in timely diagnosis (p<0.001). Graduates were also more aware than others regarding the screening procedure of an RMC and that timely diagnosis through RMC can lead to early treatment (p<0.001). The results have been summarized in Table 1. 

**Table 1:** Level of familiarity regarding RMC compared with the education level of participants RMC, routine medical checkup

Educational categories	Awareness of what an RMC is	Aware that RMC helps in timely diagnosis	Aware of what a screening procedure is	Aware that RMC involves head-to-toe exam	Aware that RMC screens all body tissues	Aware that screening tells about progressive disease	Aware that timely diagnosis can lead to early treatment
	n (%)	n (%)	n (%)	n (%)	n (%)	n (%)	n (%)
Primary	17 (51.5%)	25 (75.8%)	16 (48%)	16 (48.5%)	10 (30%)	21 (63.6%)	27 (81.8%)
Secondary	78 (78%)	92 (92%)	49 (49%)	76 (76%)	42 (42%)	73 (73%)	90 (90%)
Graduate	190 (85.2%)	212 (95%)	140 (62.8%)	164 (73.5%)	102 (45.7%)	164 (73.5%)	212 (95.1%)
p-value	<0.001	<0.001	0.037	0.006	0.237	0.488	0.015

Knowledge regarding different aspects of RMC compared in males and females 

The results of this study have shown that females had a greater overall awareness of RMCs than males (p<0.001). Females were especially aware of the fact that timely diagnosis can lead to early treatment and that RMC involves head-to-toe examination (p<0.001). These results have been summarized in Table 2.

**Table 2:** Familiarity with different aspects of RMCs compared in males and females RMC, routine medical checkup

Gender	Awareness of RMC n(%)	Aware that RMC helps in timely diagnosis n(%)	Aware of what a screening procedure is n(%)	Aware that RMC involves head-to-toe exam n(%)	Aware that RMC screens all body tissues n(%)	Aware that screening tells about progressive disease n(%)	Aware that timely diagnosis can lead to early treatment n(%)
Female	167 (85%)	187 (95%)	115 (58.7%)	156 (79.6%)	91 (46.4%)	150 (76.5%)	190 (97%)
Male	118 (74%)	142 (82%)	90 (56.3%)	100 (62.5%)	63 (39.4%)	108 (67.5%)	139 (87%)
p-value	0.007	0.018	0.645	<0.001	0.181	0.058	<0.0

RMCs and their comparison with age and educational level 

Since age was non-normally distributed, a Mann-Whitney U test was used to correlate age with awareness regarding RMC. However, it yielded insignificant results (p=0.080). Individuals were then evaluated based on whether they have ever had an RMC. The comparison of gender with whether one ever had an RMC yielded insignificant results (p=0.343). However, graduates were more likely to undergo RMCs than people educated till the primary or secondary level (p<0.001). These results are summarized in Table 3. 

**Table 3:** Level of education compared with whether participants ever had an RMC RMC, routine medical checkup

Level of education	Underwent RMC, (n(%)	Did not undergo RMC, n(%)
Primary	9 (27.3%)	24 (72.7%)
Secondary	40 (40%)	60 (60%)
Graduate	130 (58%)	93 (42%)

Reason for undergoing RMC 

**Figure 1:**
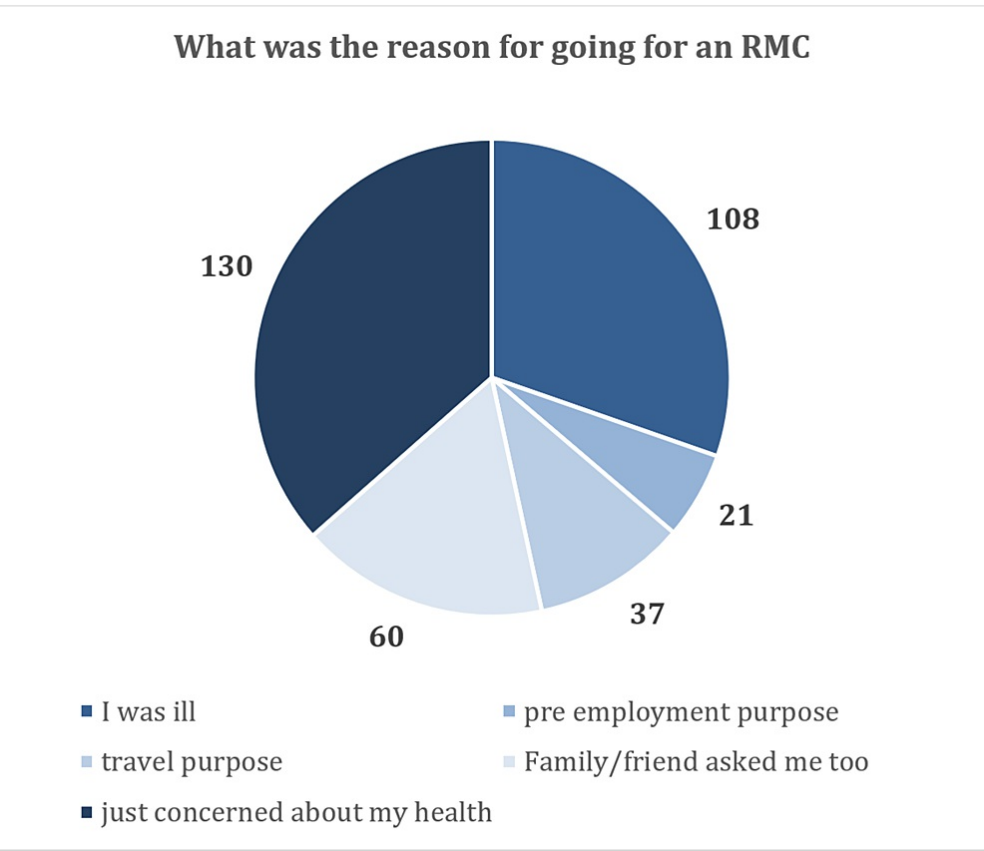
Reasons for having an RMC RMC, routine medical checkup

Moreover, participants were asked about the reasons why they underwent RMC or did not undergo RMC in their life. The most common reason for undergoing RMC was “just concerned about my health,” which was selected by 130 (36.5%) participants. This data has been summarized in Figure 1. 

Reason for not undergoing an RMC

**Figure 2:**
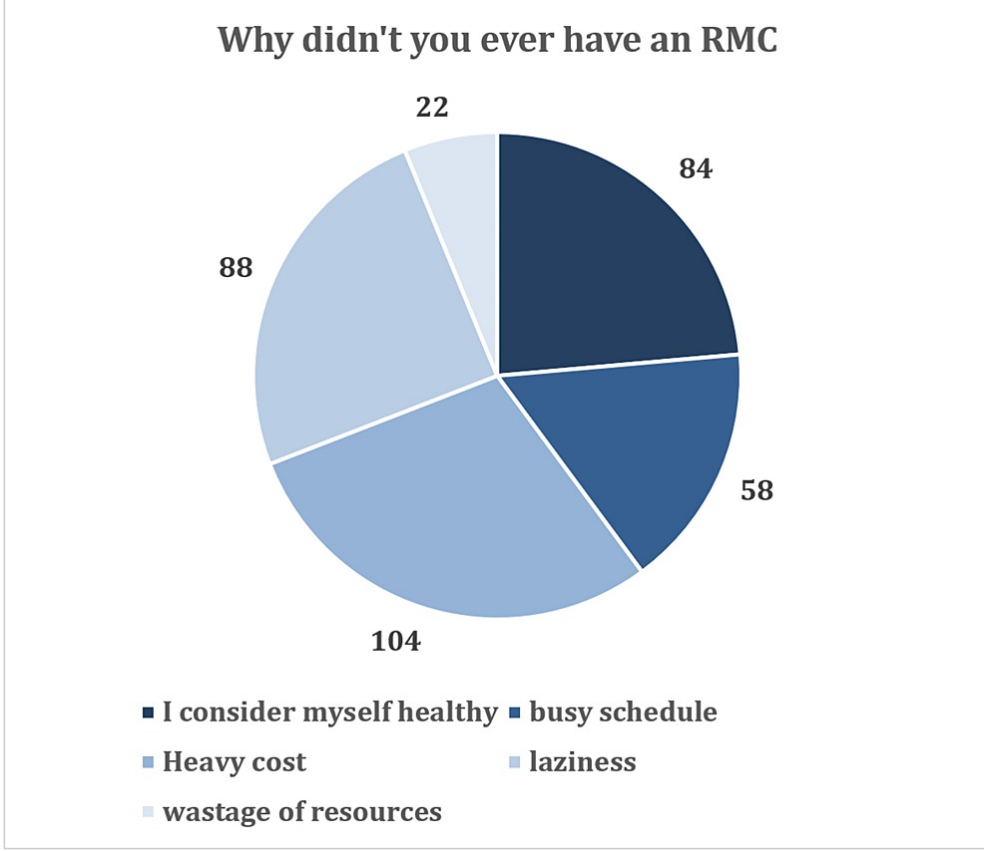
Reasons for not having an RMC RMC, routine medical checkup

Participants were also asked about their reasons for not having an RMC. The most common reason for not undergoing an RMC was “heavy cost,” selected by 104 (29.2%) participants. This data has been summarized in Figure 2.

Perceptions regarding RMCs 

Moreover, 333 (93.5%) participants thought advanced techniques are the most effective in detecting diseases, 326 (91.6%) participants thought RMCs can help in the prevention of communicable diseases, and 316 (88.8%) thought RMCs can help to reduce morbidity. However, only 239 (67.1%) participants thought that normal findings would indicate that the patient is free from disease and 272 (76.4%) thought that abnormal findings must mean that the individual is suffering from a disease. This study has also found that 346 (97.2%) individuals think RMCs should be performed, and 334 (93.8%) think that RMCs should be a part of all government employees on a half-yearly/annual basis. 

## Discussion

A total of 80% of this study’s participants said that they knew what is meant by RMCs. In a study conducted in Saudi Arabia, it was shown that 69.57% of the study participants knew about the meaning of RMCs. In the same study, it was revealed that the most common reason for having an RMC was because of "health concerns" [10]. According to this study’s participants, the most common reason for having an RMC was their ‘’concern about health.'’ Thus, in both studies, the reason for having an RMC was the same. In a study conducted in Northern Vietnam, it was shown that only 24% of people underwent an RMC [11]. This low prevalence of RMC may be due to different study settings as people living in urban areas are relatively more likely to have an RMC than people living in rural areas. The vast majority of this study’s participants believed that RMCs help in the timely diagnosis of diseases, specifically NCDs. A review of 19 randomized controlled trials showed that general health checkups are associated with increased detection of chronic diseases, such as depression and hypertension; and moderate improvements in controlling risk factors, such as blood pressure and cholesterol. This increased detection of diseases then leads to an increased usage of preventive health services by patients and improves patient-reported health outcomes [8]. 

In another study conducted in Saudi Arabia, it was concluded that having an RMC was uncommon among their study population, and the practice of having an RMC was only motivated by the existence of a diagnosed medical condition [12]. Whereas in this study, 30% of the participants believed that the reason for having an RMC was because of an illness. This shows that reasons for having an RMC are more or less similar in different countries and cultures. It is very important and significant to know the motivating factors present in the general population for having an RMC. At the same time, it is of great value to know the reasons for not having RMCs in the general population so that these factors and reasons can be appropriately addressed. 

In this study, 50% of the population had undergone an RMC in the past. In a study conducted on healthcare professionals in Nigeria, it was shown that 46% of the study population had undergone an RMC [5]. Another study in Uganda showed that 43.4% of their participants had undergone RMCs [13]. A study in the Eastern region of Saudi Arabia indicated that only 22.5% of the study population underwent RMCs [14]. This data shows that the culture of having RMCs is varied in different regions of the world. Both developed and underdeveloped countries do not have a significant percentage of their population who have undergone an RMC in their lifetime. Given the importance of RMCs, it is pertinent that healthcare systems around the world make RMCs feasible for their population. 

Thus, it is very important to understand and reveal barriers that exist in having RMCs for the general population. The most common reason revealed in this study for not having an RMC was the increased cost of these RMCs. Other reasons for not having an RMC revealed in this study include laziness, a busy routine, the belief in being healthy, and considering RMCs as a waste of resources. In a study conducted on Saudi adults, it was found that crowdedness and busy healthcare staff at primary healthcare centers along with a lack of knowledge of patients contributed to less prevalence of RMCs [15]. Another study in Iowa, US, found that decreased likelihood of having RMCs was found in those adults who were facing financial issues. An increased likelihood of having RMCs was found in those adults who have health insurance and belong to a higher-socioeconomic class [16]. In another study conducted in Northern Taiwan, it was concluded that belief in being healthy, less availability of time, the belief that screening tests are complicated, negative emotions, and negative experiences at previous screening programs were the main reasons behind not attending health screenings [17].

Furthermore, a comparison of awareness regarding different aspects of RMC was also made between males and females. Females had a greater overall awareness of RMCs than males (p<0.001). Females were especially aware of the fact that timely diagnosis can lead to early treatment and that RMC involves head-to-toe examination (p<0.001). 

Among the factors that can be linked to increased knowledge and awareness regarding RMCs, one such factor can be the level of education of an individual. In this study, the level of education was compared with awareness regarding RMCs to assess if any significant association exists between the two. It was then found that graduates were generally more aware of different aspects of RMCs (p<0.001), especially in the domains of awareness regarding what an RMC is and that RMC can help in timely diagnosis (p<0.001). Graduates were also more aware than others regarding the screening procedure of an RMC and that timely diagnosis through RMC can lead to early treatment (p<0.001). A study conducted in the Jazan region of Saudi Arabia, which has been mentioned before, showed that one of the factors associated with the level of knowledge regarding RMCs was the educational level of the study participant [5]. Thus, it can be said with quite confidence that people having a higher level of education are more likely to be aware of certain important aspects of RMCs. Another study conducted on 216 adults in Tema showed that the practice of routine medical and dental examinations was higher among adults with higher education status. Other factors that were found to increase the likelihood of increased routine medical and dental examinations was being over 50 years, being male, higher income earner, and being unmarried [18]. 

## Conclusions

Most of the participants of this study were well-educated and were students in terms of profession. The majority of the study population knew that RMCs could help in early diagnosis and treatment. Awareness regarding RMCs was linked to educational level. Females had overall better knowledge regarding RMCs than men. The most common reported reason to have an RMC was concern regarding health and the most common reported reason for not having RMC was its high cost.
